# The Relations Among Types of Parentification, School Achievement, and Quality of Life in Early Adolescence: An Exploratory Study

**DOI:** 10.3389/fpsyg.2021.635171

**Published:** 2021-03-29

**Authors:** Judyta Borchet, Aleksandra Lewandowska-Walter, Piotr Połomski, Aleksandra Peplińska, Lisa M. Hooper

**Affiliations:** ^1^ Faculty of Social Sciences, Institute of Psychology, University of Gdan´sk, Gdan´sk, Poland; ^2^ Schindler Education Center, Center for Educational Transformation, University of Northern Iowa, Cedar Falls, IA, United States

**Keywords:** school achievement, school grades, instrumental parentification, current parentification, quality of life, adolescence

## Abstract

Children who experience parentification may have trouble performing developmental tasks due to being overwhelmed by their family caregiving roles and responsibilities. Past studies have found that parentification is negatively associated with academic achievement. However, most of these studies are limited in that they are retrospective and examine the association but not the mechanisms shaping them. The aim of the study was to explore to what extent diverse types of parentification relate to academic achievement and to what extent these relations are mediated by self-reported quality of life among adolescents. The study sample was composed of Polish early adolescents (*N* = 191; age: *M* = 14.61; *SD* = 1.26). Types of parentification were measured with the Parentification Questionnaire for Youth, and quality of life was assessed with KidScreen27. School achievement was measured based on mean semester grade. We explored the associations among study variables and performed six mediation models in the planned analyses. Overall, bivariate relations were significant in a theoretically expected way, although the effect sizes for these associations were rather small. In the mediation analyses, the results showed that four of the six models were not significant. Different from previous studies, instrumental parentification was positively related to school achievement. Additionally, this positive association was mediated by adolescents’ general quality of life. Taken together, the findings were similar and different from the empirical literature base on types of parentification and select outcomes.

## Introduction

The term parentification describes the family structure when a child is placed in a parental role toward the parent(s) (Boszormenyi-Nagy and Spark, 1973; Haxhe, 2016). Carrying out parental duties by children is often highly challenging in particular when the level of family stress is high. Children also may serve other adult-like roles such as raising siblings, caring for other family members, and performing roles and tasks at a level that often exceeds the child’s age, abilities, and resources (Hooper et al., 2011a). The phenomenon of parentification is most often considered in the context of family development and the consequences of burdening children with age-inappropriate tasks implicated in their current and later development (Jurkovic, 1997; Kerig, 2005). On the other hand, there is a body of research that suggests that when a child is parentified, the resulting caregiving responsibilities may lead to an increase in maturity and the positive competences (Hooper, 2007; Kuperminc et al., 2013; Chee et al., 2014). The competence at cost template suggests the complexity of the process of parentification and the related outcomes can have both a positive and a negative impact on children’s development (Hetherington, 1999).

Thus, parentification in the family may be related to the neglect of the child’s individual and relational and bonding needs with the parent (Wasilewska and Kuleta, 2014) and serve as a benefit to the child or adolescent in various areas of functioning, including school achievement (Chase et al., 1998). Several clinical and theoretical models describe these dichotomous outcomes (see Hooper, 2017). According to some clinical models, the burden on the child is that the parent ceases to act as a regulator of the family system and the child’s instrumental and emotional life. Through the abdication of the parent role, children are forced to try to manage, regulate, and stabilize the family system, robbing the children of the ability to focus on and use their resources for their development.

Parentification has also been explained through the prism of resiliency (Hooper et al., 2008; Macfie et al., 2015). According to the theory of resilience, when some of the family caregiving duties carried out by children do not exceed their developmental capabilities, the process of role reversal may engender the child’s growth and positive outcomes. Research and clinical models suggest that the condition for positive outcomes of parentification for many children will, however, be contingent upon the parent’s recognition and appreciation for the family caregiving tasks performed (Jurkovic, 1997; Schier, 2014). Also, the positive consequences of parentification may be present in some areas of life and development while coexisting with negative ones (e.g., the adolescent may be mature, socially developed, but have problems managing her or his emotions).

The results of an increasing number of studies also indicate the benefits that can emerge when children engage in *some* caregiving responsibilities—even parental responsibilities—when these roles and responsibilities are acknowledged, appreciated, and valued by the adults in the family system. These benefits include an increase in relational competences when the child supports the parent emotionally and instrumentally, an increase in individuation and differentiation in immigrant families (Walsh et al., 2006), and psychosocial adaptation (McMahon and Luthar, 2007). These benefits are often differentiated by the type of parentification or caregiving responsibilities. The accumulated research has shown that instrumental parentification may promote a child’s competence, self-efficacy, and skills. In one study role reversal, in which adolescents instrumentally supported their parent, it contributed to their growth and self-efficacy (Mayseless et al., 2004).

Parentification is a culture-immersed phenomenon, meaning some of its antecedents, outcomes, perception, and measurement may vary across cultures (e.g., East, 2010; Gilford and Reynolds, 2010; Kuperminc et al., 2013). Therefore, international studies on parentification are highly needed (Hooper, 2014). The current study focuses on young Polish adolescents and academic achievement. Specifically, we examine how types of parentification are related to academic achievement. In order to understand this relation in the broad context of the adolescent’s situation, we examine the extent to which quality of life mediates the association.

Parentification often means that children are placed in the role of serving as a primary caregiver for the family system and its members. This can mean that the children and adolescents engage in various responsibilities and roles toward some (parents) or all of the family members. The tasks of parentified children and adolescents are developmentally inappropriate or excessive to the extent that enables them to perform their own developmental tasks (Hooper et al., 2011a).

Parentification is often differentiated by the type of caregiving activities (Jurkovic, 1997) and to whom the caregiving activities are directed (Hooper, 2009; Hooper et al., 2011b). This distinction helps to categorize the tasks that parentified children perform. Instrumental parentification consists of children performing parent-like household duties and helping care for—and in some cases raise—their siblings (Kościelska, 2007; Schier, 2014). Those may include, for example, managing family finances, earning money for the family, preparing meals, or cleaning. Instrumental parentification of adolescents may not be as easy to notice as it is in the case of younger children. The boundary between what is a fair duty that comes with age and expressive burden may not be that obvious. Therefore, it is important to remember that parentification is not only about the tasks themselves that the adolescents perform, but the fact that it is a stable pattern in their relationships with the parents and it is related to inverted family hierarchy and blurred boundaries between family members (Kerig, 2005). In this view, the housework related to parentification is not only a physical task and a duty, it is also a part of family loyalty (Byng-Hall, 2008; Haxhe, 2016).

Emotional parentification is primarily associated with the children fulfilling the emotional and social needs of their guardians (Byng-Hall, 2002, 2008; Peris et al., 2008; Schier, 2014). With this type of parentification, children can act as confidants, comforters or mediators. Both emotional and instrumental parentification are not exclusive and may coexist on various levels (Schier et al., 2015). Studies indicate that emotional parentification may be more deleterious and destructive than instrumental parentification (McMahon and Luthar, 2007; Tompkins, 2007; Byng-Hall, 2008). Ohntrup et al. (2011) contended emotional parentification is more severe because it is less explicit, overt, or possibly harder to detect than instrumental parentification. Additionally, the correlates and outcomes evidenced for these types of parentification may be different. The theoretical and empirical literature describes that instrumental parentification is not always a burden for the child, and later the adult, if the child’s contribution to family life is revealed, temporary, named, and positively assessed by the environment (Jurkovic, 1997; McMahon and Luthar, 2007; Hooper et al., 2008).

Children who are placed at risk for experiencing parentification typically encounter similar family structures and have parents and siblings diagnosed with physical and mental health disorders (Macfie and Swan, 2009). For example, family structures and parent hardships may include marital conflict (Peris et al., 2008), divorce (Wallerstein, 1985; Byng-Hall, 2008), substance abuse (Pasternak and Schier, 2014; Tedgård et al., 2019), economic hardship (Montalvo et al., 1967; Boszormenyi-Nagy and Spark, 1973), economic success (Winton, 2003), and immigrant status (Kuperminc et al., 2013; Toro et al., 2019).

Due to the complexity of the parentification process and the fact that it is embedded in culture, it is not surprising that studies present both a negative and a positive impact on children’s development and well-being. Studies show that among the negative consequences of parentification are depression, increased levels of anxiety, propensity for risky behaviors associated with stimulants, as well as eating disorders and personality disorders of the borderline and dissociation type (Cicchetti, 2004; Hooper et al., 2011a; Jankowski et al., 2013; Obsuth et al., 2014). Although there is a lack of research indicating a direct association between current parentification and school achievement, it can be assumed on the basis of the literature that children who present with developmental challenges may have problems with learning. Additionally, it could be that these children may be living in a family context where parentification exists. On the other hand, constructive parentification may help adolescents learn efficient task management and thus facilitate school achievement or shape tendencies for compulsive overworking to fulfill tasks at home and school. Moreover, culture-specific factors may shape the relation between parentification and its bimodal outcomes (e.g., Gilford and Reynolds, 2010; Kuperminc et al., 2013; Burton et al., 2018).

While there are a lack of studies investigating the direct relationship between an adolescent’s general quality of life and parentification, there are studies on parentification in association with constructs that are similar or related to the quality of life (e.g., well-being, life satisfaction, and positive and negative affect). Parenting behavior, including parentification, may predict family member’s well-being (Burton et al., 2018). Parentification is generally negatively related to well-being, but those relations can be differentiated by the type of parentification. Parent-focused and sibling-focused parentification are negatively related to well-being while perceived benefits of parentification present positive association with satisfaction with life (Hooper et al., 2014). Moreover, negative indicators of emotional well-being were found to be linked to both emotional and instrumental parentification in the sample of Polish 16-year-olds (i.e., anger and depressive mood, no correlation with positive mood; Żarczyńska-Hyla et al., 2019). Including quality of life in studies on parentification can help contextualize the results and investigate bimodal consequences of parentification (see the study on parentification, psychopathology, and well-being; Hooper et al., 2014).

Parentification may have relevance to academic achievement too. Parents may burden their children with meeting their high expectations for academic achievement and success (Winton, 2003; Haxhe, 2016). In this case, parentification is often emotional in its nature, as the child appeases the parent by satisfying her or his unmet need for achievement (Winton, 2003). Emotional parentification is often more difficult to detect, and similar to other role reversal situations, parentification can have short- and long-term consequences for the development of the child (Chase et al., 1998). Experiencing parentification, for example, caregiving for a parent who is disabled, substance-dependent, or experiencing a medical or emotional crisis is a very difficult situation for children and thus they often feel shame, isolation, and stigma. This may result in “secret keeping” related to parentification and family functioning, which in turn could prevent children from talking to anyone about what is happening in the family. Such conviction leads to the inability to seek help from people other than family members (Tedgård et al., 2019).

Although there is evidence of the association between parentification and outcomes over the course of a child’s lifespan, there is a lack of research on the consequences of the adult task load on the functioning of the child in school (see, for review, Macfie et al., 2015). While investigating the consequences of parent-child role reversal on child and adolescent development at its various stages, problems with learning and peer relationships occurred most often at the school age (Macfie et al., 2015). Studies by Baldwin and colleagues (Baldwin et al., 1982) show that an imbalance in parent-child relationships is associated with lower level of academic competence (as measured by teacher’s and parent’s assessment) among youth. They contend that the imbalance where there is clear dominance of the child in the parent-child relationship is similar to role reversal seen in parentification. Research on adolescents taking care of parents diagnosed with various mental disorders showed that one-fifth of these adolescents present with school-related problems (Dearden and Becker, 2004), and half of them report difficulties with homework (Thomas et al., 2003). Other studies found that the impact of an imbalance in parent-child relationships on grades can also be observed (e.g., an association between poor grades and the length of time children were caregivers for parents; Cree, 2003). The author explains the results of the study, stating that a child caring for a sick parent has no time for other activities, including homework, and does not receive support from the parent in preparing homework.

One of the few studies in which a direct negative association between parentification and academic outcomes was carried out by Chase et al. (1998). They found that fulfilling the parental role by a child may disrupt the course of high school education and later result in lower academic outcomes among college students. The study, however, was limited in that it required emerging adults to recall their parentification experience retrospectively. Additionally, parentification was measured by a single score (i.e., no measure of the types of parentification). On the other hand, a study by Gilford and Reynolds (2010) showed that Black American emerging adults who grew up in single-parent households and with a history of instrumental and emotional parentification were successful in college and demonstrated a positive outlook, strength, and resilience. Many of the interviewed women were able to use the difficulties from their childhood to motivate themselves to complete college and to pass that motivation and inspiration onto their siblings (Gilford and Reynolds, 2010).

In the Polish cultural context, there is one recent study (Żarczyńska-Hyla et al., 2019) that examined whether Polish adolescents attending different types of schools have different experiences related to the burden of reversing the roles in the family. Results in this study found no differences in emotional and instrumental parentification and study outcomes, although young people attending vocational schools perceived their situation in the family as being more unfair in terms of the burden of tasks and responsibilities of adults as compared to young people attending other types of schools (technical secondary school, high school). The results are difficult to interpret; however, the authors suggest that parentification experienced in the family may be associated with the choice of school by young people. Young people choosing vocational schools, due to their family situation, could receive less support from their parents in school education and choose at the next stage the apprenticeship, with the possibility of starting full-time work at the age of approximately 18 years (vocational schools in Poland are the last stage of education, usually without the possibility of continuing higher education).

School learning is a task that requires an appropriate level of development—not only cognitive—but also emotional and social. School problems may be a symptom presented by the child as a family delegate, pointing toward other difficulties derived from the functioning of the family as a system (Chase et al., 1998). Effective functioning at school requires children to focus on themselves and school tasks. Consistent parental support aids in this learning context and process. Unfortunately, the resources of parentified children are invested in and directed toward meeting the needs of others: family members, parents, and siblings (Chase et al., 1998; Siskowski, 2006). Children affected by parentification may show deficits in various areas of development, to varying degrees of severity, depending on whether they performed instrumental tasks and emotional tasks, support their parents and siblings, or satisfy their need for success. On the other hand, they may present with confidence, resilience, and high-level task management and coping skills. Instead of underachieving at school, parentified children and adolescents may do well at school and experience depression, anxiety, and low well-being. Therefore, the studied relations between parentification characteristics and school achievement have been put into the broad context of adolescent’s general quality of life. Thus, the aim of the study was to explore the relation between types of parentification and school achievement and the extent to which quality of life mediates the association.

## Materials and Methods

### Procedure

Prior to beginning the study, the approval from the University’s IRB was received. The study was conducted during the 2017/2018 school year (September to November). There were two public schools invited to join the study. These schools have participated in a broader research project conducted by the University of Gdańsk and the school’s administration and teachers offered their assistance with the recruitment and administration phases of the study. The teachers helped to administer the informed consent forms to students’ parents or legal guardians. Prior to the administration of the survey, adolescents provided their informed assent for the study. After a brief introduction, the students completed paper-and-pencil questionnaires during one of their classes. The study procedures lasted approximately 25–30 min. After the students completed the survey, they were thanked for their participation.

### Participants

The study was performed in two public schools located in two districts of the city of Gdańsk, Poland. There were 191 adolescents who participated in the study. Every class member was invited to join the study but only the students whose parents provided consent and had siblings participated. Girls constituted 55% (*n* = 105) of the sample, boys 44.5% (*n* = 85), and 0.5% (*n* = 1) of the study participants did not provide information on their gender. The participants were aged 12–18 years old, with a median of 14 (*M* = 14.61; *SD* = 1.26). All of the participants had siblings and 90.1% of them lived with both their parents. The participants were diverse based on their socioeconomic background. On a scale from 1 to 10, the mean family socioeconomic status was *M* = 6.6; *SD* = 1.68.

### Measures

The study administered two questionnaires (PQY-Parentification Questionnaire for Youth, Borchet et al., 2020a; Polish version of KidScreen-27, Mazur et al., 2008) and a demographic information sheet.

#### Demographic Information Sheet

The demographic information sheet asked participants to respond to several questions about their background. We collected information about the participant’s gender (considered bivariate: female/male) and age (considered continuously). Information about their families such as socioeconomic status (SES) and family structure was also captured in the demographic sheet. Response options for family SES used a Cantril ladder ranging from 1 (*the poorest families in Poland*) to 10 (*the richest families in Poland*). The participants were also asked about their family structure (i.e., living with both parents, living with mother; living with father, living with a mother and her partner, living with a father and his partner). Students also reported information about their last school year’s final mean grade.

#### Parentification

Parentification Questionnaire for Youth (PQY; Borchet et al., 2020a) is a measure developed for adolescents that captures the multidimensional nature of parentification. The questionnaire consists of 26 items rated on a 1 (*never true*)- to 5 (*always true*)-point Likert-type scale. The scale consists of four subscales (emotional parentification toward parents, instrumental parentification toward parents, sense of injustice, and satisfaction with the role) and two subscales for adolescents who have siblings (i.e., instrumental parentification toward siblings and emotional parentification toward siblings). Scores are calculated as the mean of the ratings for the subscale items. The questionnaire does not provide a total score (Borchet et al., 2020a). Reliability for the subscale scores was sound (i.e., Cronbach’s *α* from 0.70 to 0.80).

#### School Achievement

This variable was operationalized as arithmetical mean grade for all the final grades obtained in all the subjects at the end of the former school year (it is reported on their yearly certificate of class completion). Grades that students can get in Poland vary from 1 to 6, with 6 being the best grade possible and 2 is the lowest grade that allows passing a class. In our sample, the mean grade varied from 2 to 6, with a median of 4.52 (*M* = 4.47; *SD* = 0.79). The mean final grades were student self-reported. The current study used mean final grades from the 2016/2017 school year.

#### Quality of Life

To assess the student’s overall perception of their lives, the Polish adaptation of KidScreen-27 was used (Mazur et al., 2008). It is a health-related quality of life measure that was developed in 13 countries by the KIDSCREEN Research Group (Robitail et al., 2007). The scale consists of 27 items referring rated from 1 to 5, with 1 meaning “*never*” and 5 meaning “*always*.” The KidScreen-27 measures five dimensions of quality of life, which are physical well-being, psychological well-being, parent relationships and autonomy, social support and peers, and school environment. The scale provides a total score that is generated by summing up all item responses (see Berman et al., 2016). The reliability coefficient for the quality of life total score was sound (Cronbach’s *α* = 0.777).

### Data Analytic Procedures

In order to explore the association between types of parentification and school achievement, and the mediating role of the quality of one’s own life in this relation, an analysis of direct and indirect effects in SEM models was carried out using the Amos 25 package. Model fit was judged using the comparative fit index (CFI), goodness-of-fit index (GFI), chi-square value (CMIN), as well as root mean square error of approximation (RMSEA). With respect to the fit indices, GFI and CFI values greater than 0.90 were considered as well-fitted (Konarski, 2010). RMSEA values lower than or equal to 0.08 indicate acceptable fit (Hu and Bentler, 1999). Ideally, CMIN would be statistically insignificant, but this value is sensitive to the sample size (Konarski, 2010). Full mediation was recognized consistent with Baron and Kenny’s (1986) criteria.

## Results

Descriptive results, Pearson correlations between variables, and the reliability of the measures were assessed with Statistical Package for Social Science (SPSS) 24. They are presented in Table 1.

**Table 1:** Summary statistics and correlations between study variables (*N* = 191).

Variable	*M*	*SD*	Min	Max	*K-S*	α	1	2	3	4	5	6	7	8
SA	4.47	0.79	2.00	6.00	0.064	-	1							
QoL	93.59	17.03	37.00	130.00	0.052	0.777	0.172^*^	1						
IPTP	2.74	0.80	1.00	5.00	0.083^**^	0.710	0.176^*^	0.209^*^	1					
EPTP	1.82	0.62	1.00	3.75	0.156^**^	0.692	−0.070	−0.010	0.228^**^	1				
SI	2.16	0.92	1.00	4.60	0.135^**^	0.803	−0.160^*^	−0.528^**^	−0.228^**^	0.098	1			
SWR	3.43	0.93	1.00	5.00	0.095^**^	0.756	0.173^*^	0.649^**^	0.398^**^	0.033	−0.602^**^	1		
IPTS	2.45	0.89	1.00	5.00	0.089^**^	0.700	0.206^**^	0.156^*^	0.411^**^	0.240^**^	−0.001	0.282^**^	1	
EPTS	2.25	0.79	1.00	4.50	0.134^**^	0.767	0.138	0.138	0.250^**^	0.311^**^	0.072	0.225^**^	0.611^**^	1

SA, school achievement; QoL, quality of life; IPTP, instrumental parentification toward parents; EPTP, emotional parentification toward parents; SI, sense of injustice; SWR, satisfaction with the role (played in the family system); IPTS, instrumental parentification toward siblings; EPTS, emotional parentification toward siblings; *K*-*S*, Kolmogorov-Smirnov test;α, Cronbach’s alpha coefficient; ^*^p < 0.05; ^**^p < 0.01.

### Zero-Order Correlation Analysis

The correlation matrix (see Table 1) revealed statistically significant associations between school achievement, quality of life (*r* = 0.17, *p* < 0.05), and most of the PQY subscales, although the effect sizes of these associations were low. Instrumental parentification toward parents (*r* = 0.18, *p* < 0.05), instrumental parentification toward siblings (*r* = 0.21, *p* < 0.01), and satisfaction with family role (*r* = 0.17, *p* < 0.05) were positively related to school achievement. Sense of injustice was negatively related to school achievement (*r* = −0.16, *p* < 0.05). Emotional parentification, neither focused on the parents nor focused on the siblings, was associated with school achievement. Quality of life was positively related to satisfaction with the family role (*r* = 0.65, *p* < 0.01) and negatively associated with sense of injustice (*r* = −0.53, *p* < 0.01).

### Mediation Analyses

**Figure 1:**
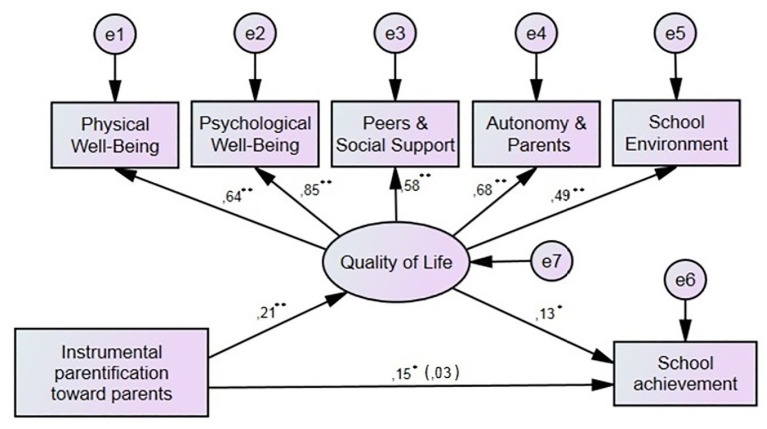
The mediating effect of quality of life on the relation between instrumental parentification toward parents and school achievement. ^*^p < 0.05; ^**^p < 0.01.

**Figure 2:**
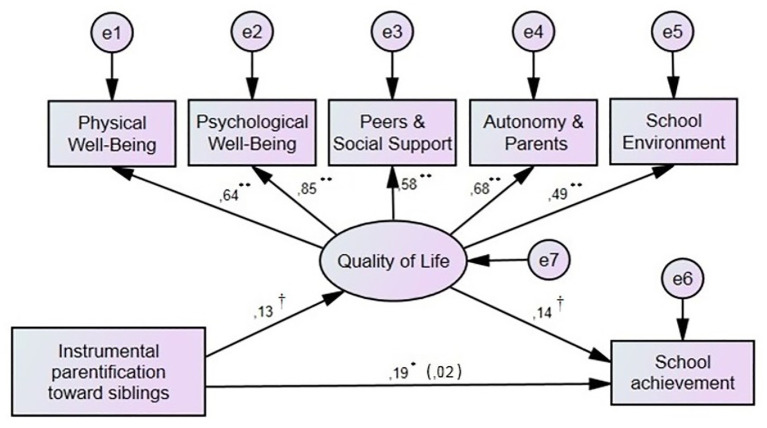
The mediating effect of quality of life on the relation between instrumental parentification toward siblings and school achievement. ^†^*p* < 0.1; ^*^p < 0.05; ^**^p < 0.01.

Six mediation models were tested, with school achievement serving as the dependent variable, quality of life was the mediator, and one of the six parentification dimensions served the role of the independent variable. The results showed acceptable fitted and interpretable models in two cases related to instrumental parentification. Four of the six tested models did not present satisfactory model fit (see Table 2). The analysis revealed that the level of instrumental parentification toward both parents (*B* = 0.15, *p* = 0.001) and siblings (*B* = 0.19, *p* = 0.001) was significantly positively related to school achievement. When quality of life was added to these two models, the relation between instrumental parentification, both toward parents and siblings, and academic achievement was statistically insignificant (see Figures 1, 2). The analyses of direct and indirect effects for both models indicated full mediation (Baron and Kenny, 1986; see Table 3).

**Table 2:** Model fits for tested mediation models by the independent variable.

Independent variable	CMIN	RMSEA	GFI	CFI
IPTP	**110.751 (39), *p*** = **0.01**	**0.071, *p*** = **0.017**	**0.925**	**0.879**
EPTP	101.388 (39), *p* = 0.00	0.091, *p* = 0.001	0.919	0.889
SWR	129.963 (39), *p* = 0.00	0.089, *p* = 0.001	0.910	0.884
SI	190.613 (39), *p* = 0.00	0.101, *p* = 0.001	0.869	0.803
IPTS	**140.731 (39), *p*** = **0.01**	**0.083, *p*** = **0.020**	**0.910**	**0.836**
EPTS	142.488 (39), *p* = 0.00	0.095, *p* = 0.001	0.907	0.830

IPTP, instrumental parentification toward parents; EPTP, emotional parentification toward parents; SWR, satisfaction with the role (played in the family system); SI, sense of injustice; IPTS, instrumental parentification toward siblings; EPTS, emotional parentification toward siblings. Bold values stand for well-fitted models.

**Table 3:** Direct and indirect effect in the tested mediation models.

Hypothesis	Direct effect	Indirect effect	Results
IPTP -> QoL -> SA	0.148^*^	0.03	Full mediation
IPTS -> QoL -> SA	0.187^*^	0.02	Full mediation

SA, school achievement; QoL, quality of life; IPTP, instrumental parentification toward parents; IPTS, instrumental parentification toward siblings.

* *p* < 0.05.

## Discussion

The study aimed to explore the relation between parentification and school achievement in the context of adolescent’s quality of life. First, we explored bivariate relations between study variables. Second, we performed mediation analyses. The study results indicated that among the six tested mediation models, only two of them were well-fitted and interpretable (i.e., models with instrumental parentification toward parents and instrumental parentification toward siblings as independent variables). Two important findings emerge from this study: (a) instrumental parentification toward parents and instrumental parentification toward siblings were positively related to school achievement and (b) these relations were mediated by adolescent’s general quality of life. Not surprisingly, as instrumental parentification can lead to positive outcomes, in our sample, it was positively related to school achievement. Moreover, a positive opinion about one’s life and its aspects, along with the circumstances of high level of instrumental parentification toward parents and siblings, may contribute to better fulfillment of other instrumental tasks such as educational tasks and outcomes. From the experience of instrumental parentification, the adolescents could have learned abilities useful at school such as how to manage their tasks effectively, shape task-oriented coping strategies (Hooper et al., 2008; Thastum et al., 2008), and build their self-efficacy (Mayseless et al., 2004).

The current findings are in line with the idea that one risk factor may not be destabilizing enough for the occurrence of any disturbance in the functioning of an individual. It is rather the coexistence of several risk factors that can cause maladjustment. For example, it has been shown that the action of one or two risk factors has a slightly negative effect on one’s functioning, while when three or more factors are operating, the impact is already significant (Kumpfer, 1999; Masten and Powell, 2003; Greenberg, 2006). Additionally, according to Rutter (1987), the occurrence of both risk and protective factors is more related to turning points in human life than to factors as such. In other words, it is more important which processes are triggered by a certain risk factor than that factor itself. Nevertheless, the authors of the research on positive adaptation point out that the lack of disturbances at the behavioral level does not mean freedom from problems related to mental health (Luthar et al., 2000; Luthar and Zelazo, 2003). The results of some studies show that adults who coped with adversities in childhood and successfully function in social roles in adulthood are not fully happy and satisfied with their lives (Luthar and Zigler, 1991). It can be presumed that adolescents who perceive their life negatively may also experience additional adversities other than instrumental parentification that can disrupt their development and school achievement (e.g., bullying, low self-esteem). The instrumentally parentified adolescents that took part in the study achieved well at school, but their success still may be a competence gained at the cost of other domains (see Hetherington, 1999). Both Jurkovic (1997) and Winton (2003) contend children and adolescents who experience parentification may present tendencies for overachieving, workaholism, and perfectionism, and those positive outcomes could mask the negative outcomes or go underreported.

Our study did not support the previous findings indicating that emotional parentification is negatively related to school achievement (see Chase et al., 1998; Siskowski, 2006; East, 2010). The lack of this effect may stem from the sample characteristics (i.e., urban and high SES). First, the family SES in the studied sample was rather high. Second, the mean scores achieved in the subscales emotional parentification toward parents and emotional parentification toward siblings were low. Also, the emotional parentification toward parents had a lower score range than other parentification variables. Therefore, students that took part in this study could have been specific and come from families where the level of emotional parentification was rather low. On the other hand, the level of emotional parentification could have been underreported in this sample [e.g., due to adolescents’ defense mechanisms aimed to present a positive image of the parent despite the adversities (see Schier et al., 2015)]. The underreporting of emotional parentification in this sample could also be rooted in the culture. Polish culture holds a significant power distance, also in terms of family hierarchy and loyalty (Hofstede et al., 2010). Combined with the tendency to keep family problems in the close circle of relatives in order to protect family reputation, along with reluctance to report family violence (Ipsos Loyalty, 2014), those may be factors that decrease the Polish adolescent’s willingness to share their emotional parentification experience.

Similar to previous studies, the sense of injustice, as well as the satisfaction with the family role, showed associations with adolescents’ school achievement. The more the adolescents perceived their family roles as unfair, the worse grades at school they had (comp., Jurkovic et al., 2005). Accordingly, the more they were satisfied with their family role, the more school achievement they had (comp., Burton et al., 2018).

### Limitations and Future Directions

The findings of this study have to be seen in the light of some limitations. First, the study employed cross-sectional assessment. To address this limitation, it is important to carry out similar studies, including a longitudinal study in the future. This would enable clarification if, while growing up, the instrumentally parentified adolescents who perceive their lives positively are able to have positive school achievements over time. Typically, as youth age, adolescents are assigned more tasks, responsibilities, and developmental roles related to their age. Another design limitation is the fact that the information about last semester’s mean school grade could have been distorted in some cases due to the fact that it was self-reported by the participants. Future studies could employ more objective source of information on the school achievement (e.g., retrieve them from the school archives). Also, adding additional informants to the study (e.g., headteachers’ assessments) might have given more insight into the child’s school performance (see Bauer et al., 2013). The sample might have been biased. Specifically, the study participants lived in the big city of Gdańsk (as measured by Statistics Poland, 2019). Future studies on the effect of current parentification on adolescents’ life should also include the participants of various backgrounds, for example, rural (see American study by Hooper et al., 2012), as well as living in small towns and middle-sized cities and family SES-diverse. That would increase the generalizability of the findings on the broad population of Polish adolescents as almost 40% of the total Polish population lives in rural areas (World Bank, World Development Indicators, 2019). Moreover, controlling for parental employment status (e.g., full time, part time, and more than full time) could provide important insight into the studies on instrumental parentification, as parents working long hours is one of the factors that may put their children in charge of the house (see Schier, 2014). This factor could have been very relevant to our sample as the participants came from rather high-SES families where parents could have been highly engaged in their careers and often absent from home. Therefore, their children could be in charge of many house chores. Also, it would be interesting to consider the adolescent’s motives for achieving good grades in future studies, for example, whether it was self-motivated, aimed to impress others or satisfy parent’s needs (see Winton, 2003; Haxhe, 2016). Another study limitation may be related to the use of a measure in a state of its infancy. Further studies should examine how does the PQY (Borchet et al., 2020a) act in various samples in order to make sure if future refinement of the questionnaire may be beneficial.

## Conclusion

The current study casts new light into the studies on the current experience of parentification in Polish adolescents. In contrast to the previous studies (Chase et al., 1998; Siskowski, 2006), the level of instrumental parentification was positively related to academic achievement. Associations with school achievement were also observed for the sense of injustice and the satisfaction with family role, which underlines the importance of how adolescents perceive one’s family role in the outcomes of parentification. Surprisingly, emotional parentification was not related to school achievement. We believe that the relation between parentification dimensions and school achievement seems to be complex and should be interpreted with caution and in a broader context (e.g., quality of life and adolescent’s motivation for school achievement). This result underlines how important it is for the parentified children and adolescents to belong to environments facilitating their development and to be satisfied with at least some areas of their lives. Those can enable them to perceive their situation positively and foster their development, also in the context of school achievement, despite the task overload and adversities they face at the family home. This buffering role may be served, for example, by the adolescent’s good health condition, the school environment, friends, hobbies, and sibling relationships or other family ties (see Chee et al., 2014; Borchet et al., 2020b).

## Data Availability Statement

The datasets generated for this study are available on request to the corresponding author.

## Ethics Statement

The studies involving human participants were reviewed and approved by Komisja Etyki ds. Projektów Badawczych przy Instytucie Psychologii UG. Written informed consent to participate in this study was provided by the participants’ legal guardian/next of kin.

## Author Contributions

JB designed and executed the study. AL-W collaborated with the design of the study. JB and AL-W wrote the manuscript. LH reviewed, revised, and edited the manuscript. PP analyzed the data. AP collaborated with editing of the manuscript. All authors contributed to the article and approved the submitted version.

## Conflict of Interest

The authors declare that the research was conducted in the absence of any commercial or financial relationships that could be construed as a potential conflict of interest.
